# The requirement for calcification differs between ecologically important coccolithophore species

**DOI:** 10.1111/nph.15272

**Published:** 2018-06-19

**Authors:** Charlotte E. Walker, Alison R. Taylor, Gerald Langer, Grażyna M. Durak, Sarah Heath, Ian Probert, Toby Tyrrell, Colin Brownlee, Glen L. Wheeler

**Affiliations:** ^1^ Marine Biological Association Plymouth PL1 2PB UK; ^2^ School of Ocean and Earth Science University of Southampton Southampton SO14 3ZH UK; ^3^ Department of Biology and Marine Biology University of North Carolina Wilmington Wilmington NC 28403‐591 USA; ^4^ Station Biologique de Roscoff Place Georges Teisser 29680 Roscoff France

**Keywords:** calcification, coccolithophore, *Coccolithus braarudii*, *Emiliania huxleyi*, phytoplankton

## Abstract

- Coccolithophores are globally distributed unicellular marine algae that are characterized by their covering of calcite coccoliths. Calcification by coccolithophores contributes significantly to global biogeochemical cycles. However, the physiological requirement for calcification remains poorly understood as non‐calcifying strains of some commonly used model species, such as *Emiliania huxleyi*, grow normally in laboratory culture.
- To determine whether the requirement for calcification differs between coccolithophore species, we utilized multiple independent methodologies to disrupt calcification in two important species of coccolithophore: *E. huxleyi* and *Coccolithus braarudii*. We investigated their physiological response and used time‐lapse imaging to visualize the processes of calcification and cell division in individual cells.
- Disruption of calcification resulted in major growth defects in *C. braarudii*, but not in *E. huxleyi*. We found no evidence that calcification supports photosynthesis in *C. braarudii*, but showed that an inability to maintain an intact coccosphere results in cell cycle arrest.
- We found that *C. braarudii* is very different from *E. huxleyi* as it exhibits an obligate requirement for calcification. The identification of a growth defect in *C. braarudii* resulting from disruption of the coccosphere may be important in considering their response to future changes in ocean carbonate chemistry.

## Abstract 2

See also the Commentary on this article by https://doi.org/10.1111/nph.15409.

## Introduction

Coccolithophores (Calcihaptophycidae) are globally abundant, single‐celled marine phytoplankton characterized by the production of elaborate calcite platelets (coccoliths). These are produced in an intracellular compartment (coccolith vesicle) and secreted to the cell surface, where they are arranged extracellularly to form a coccosphere (Brownlee & Taylor, 2003; Marsh, 2003; Taylor *et al*., 2017). As a result of their global prevalence and ability to form vast blooms (Westbroek *et al*., 1993), coccolithophores are estimated to be responsible for up to 10% of global carbon fixation (Poulton *et al*., 2007) and are major producers of oceanic biogenic calcium carbonate. Calcification by coccolithophores contributes to a rain of calcite from surface waters to depth, which can remineralize and contribute to a vertical alkalinity gradient in the water column (Milliman, 1993) or form vast sedimentary deposits on the ocean floor (Thierstein *et al*., 1977). In addition, sinking coccoliths ballast particulate organic matter, enabling the transfer of organic carbon to depth (Ziveri *et al*., 2007). Consequently, coccolithophores are crucial contributors to ocean biogeochemical cycles and much research has focused on how calcification may be impacted by future changes in ocean carbonate chemistry (Riebesell *et al*., 2000; Rost & Riebesell, 2004; Ridgwell *et al*., 2009; Meyer & Riebesell, 2015).

Given the biogeochemical importance of calcification, it is surprising that the ecological and physiological reasons underlying coccolith production remain uncertain (Tyrrell & Merico, 2004; Monteiro *et al*., 2016). Several species exhibit the ability to grow without coccoliths in laboratory culture, most notably *Emiliania huxleyi* and *Chrysotila carterae* (formerly *Pleurochrysis carterae*) (Paasche, 2001; Marsh, 2003). The diploid heterococcolith‐bearing life stages of these species are invariably fully calcified on initial isolation, although many strains that have been maintained in laboratory culture for several years are only partially calcified or have lost the ability to calcify entirely (Paasche, 2001; Marsh, 2003). Non‐calcifying strains of *E. huxleyi* are genetically diverse, suggesting that this characteristic is not restricted to a single lineage or morphotype (Kegel *et al*., 2013; Read *et al*., 2013). These observations suggest that calcification is not essential for the growth of coccolithophores, at least when they are maintained in laboratory culture. In turn, this finding has important implications for our understanding of coccolithophore ecology, especially when we consider the potential impact of future changes in ocean carbonate chemistry on the calcification process (Riebesell *et al*., 2000).

However, there is currently little experimental evidence examining the requirement for calcification in other coccolithophore species, and there is evidence suggesting that the commonly used laboratory models *E. huxleyi* and *C. carterae* may not be typical of all coccolithophores. For example, the large, heavily calcified species, such as *Calcidiscus leptoporus* and *Coccolithus braarudii*, which contribute significantly to calcification in our global oceans (Daniels *et al*., 2014), always appear to be fully calcified in exponentially growing diploid cultures. In addition, there are some indications of mechanistic differences in the process of calcification between coccolithophores. For example, several species, including *C. braarudii*, exhibit a requirement for silicon (Si) in the calcification process, whereas this requirement is entirely absent from other species, such as *E. huxleyi* (Durak *et al*., 2016). It is also likely that coccolith production fulfils multiple roles within coccolithophores, which may differ between species (Monteiro *et al*., 2016). In the light of these contrasts, it is essential to question whether these species exhibit an obligate dependence on calcification for cellular fitness that relates to important differences in either the process or the function of calcification between coccolithophore lineages.

The availability of non‐calcifying strains of *E. huxleyi* has been used to assess the potential role of calcification in this species. Surprisingly, the absence of calcification, in either non‐calcifying strains or by depletion of Ca^2+^ in calcifying strains, has little obvious impact on *E. huxleyi* physiology in laboratory cultures, with no reduction in growth rate or photosynthesis (Herfort *et al*., 2004; Trimborn *et al*., 2007; Leonardos *et al*., 2009). Although calcification in *E. huxleyi* commonly occurs at a similar rate to photosynthesis, current evidence does not support a role for calcification as a carbon‐concentrating mechanism in this species (Herfort *et al*., 2002; Trimborn *et al*., 2007; Leonardos *et al*., 2009; Bach *et al*., 2013). There is also no evidence to suggest that calcified *E. huxleyi* cells are better protected from zooplankton grazing (Harris, 1994) or viral infection (Wilson *et al*., 2002). Several studies have also indicated that the coccosphere does not contribute to the protection from photoinhibition (Nanninga & Tyrrell, 1996; Trimborn *et al*., 2007), although recent evidence indicates that the non‐calcifying strains may be more sensitive to UV radiation and grow less well under natural light (Xu *et al*., 2016). Given that there are few clear physiological differences between calcifying and non‐calcifying *E. huxleyi* strains, evidence in support of the many proposed roles of calcification remains limited.

The absence of non‐calcifying strains has precluded similar investigations into the requirement for calcification in most other coccolithophore species. However, it is possible to disrupt calcification in coccolithophores experimentally by using a range of different techniques. For example, *E. huxleyi* cells grown at 0.1 mM Ca^2+^ in artificial seawater media are non‐calcified, whereas cells grown at 1 mM Ca^2+^ produce incomplete coccoliths with extensive malformations (Herfort *et al*., 2002, 2004; Trimborn *et al*., 2007; Leonardos *et al*., 2009). At 1 mM Ca^2+^, *E. huxleyi* cells grow normally, although cells grown at extremely low Ca^2+^ (< 0.1 mM) exhibit minor growth defects (Trimborn *et al*., 2007; Mackinder *et al*., 2011). *Chrysotila haptonemofera* (formerly *Pleurochrysis haptonemofera*) exhibited reduced calcification at 5 mM Ca^2+^ and growth was negatively impacted at concentrations of 0.5 mM Ca^2+^ (Katagiri *et al*., 2010). As Ca^2+^ is essential for many cellular processes, most notably cell signalling, extreme Ca^2+^ depletion could potentially affect many wider aspects of cell physiology. An alternative mechanism to inhibit calcification is the application of bisphosphonates, such as 1‐hydroxyethane 1,1‐diphosphonic acid (HEDP), which inhibit calcification through their ability to chelate metal ions and prevent the growth of calcium carbonate crystals. HEDP has been used extensively in other calcified organisms (e.g. fresh water algae (Heath *et al*., 1995) and corals (Yamashiro, 1995)) and also inhibits calcification in the coccolithophores *E. huxleyi* (1 mM) (Sekino & Shiraiwa, 1994) and *C. carterae* (0.5 and 1 mM) (Asahina & Okazaki, 2004). In addition, we have recently identified that the Si analogue germanium (Ge) may be used to disrupt calcification in the coccolithophore species that exhibit a requirement for Si in coccolith production (Durak *et al*., 2016).

In this study, we have examined whether the ecologically important species *C. braarudii* exhibits an obligate dependence on calcification for growth. *C. braarudii* and the closely related species *C. pelagicus* are abundant in temperate and subarctic regions, respectively, of the Atlantic and Pacific oceans, and their large coccoliths contribute significantly to the sedimentary deposition of calcite from the photic zone (Ziveri *et al*., 2004; Daniels *et al*., 2016; Tsutsui *et al*., 2016). Although *C. braarudii* strains have been maintained in laboratory culture for many years, non‐calcifying diploid strains have not been identified. Previous experiments to manipulate calcification in coccolithophores have primarily utilized a single disruption technique, limiting the ability to identify non‐specific impacts of the treatment on other cellular functions. We have therefore employed multiple methodologies to disrupt calcification to ensure that our observations are primarily a result of a defect in coccolith production. We show that disruption of calcification using four different methods leads to inhibition of growth in *C. braarudii*. We do not find evidence for a link between calcification and photosynthetic function, but find that cell division is inhibited in cells that are unable to form a complete coccosphere.

## Materials and Methods

### Algal strains and culture conditions

*Coccolithus braarudii* (PLY182g) (formerly *Coccolithus pelagicus* ssp. *braarudii*) and *E. huxleyi* (CCMP1516) were grown in filtered seawater (FSW) with added f/2 nutrients (Guillard & Ryther, 1962) and added [dSi] 10 μM (unless specified). Cells were grown in triplicate batch cultures, incubated at 15°C and illuminated with 65–75 μmol photons m^−2^ s^−1^ in a 16 h : 8 h, light : dark cycle.

### Cell growth and discarded coccoliths

Cells were counted using light microscopy and a Sedgewick–Rafter counting chamber. Growth rates (d^−1^) were determined from the initial and final cell densities (*N*_t0_, *N*_t1_) using the formula: SGR = [log_e_(*N*_t1_) − log_e_(*N*_t0_)]/*t*. Discarded coccoliths were also counted by light microscopy. We did not discriminate between regular and aberrant coccoliths for this count. Statistics were completed using sigmaplot v.13.0 software (Systat Software Inc., London, UK).

### Disruption of calcification

#### Low Ca^2+^

To control the availability of Ca^2+^, Harrison's broad‐spectrum artificial seawater (ASW; Harrison *et al*., 1980) was used, with the addition of H_2_SeO (final concentration, 5 nM) and omission of CaCl_2_. The addition of H_2_SeO was made as it has been shown previously that *E. huxleyi* requires selenium for growth (Danbara & Shiraiwa, 1999). Before treatment, *C. braarudii* and *E. huxleyi* cells were acclimated at 10 mM Ca^2+^ ASW for several generations (> 2 wk) and then treated with a range of Ca^2+^ concentrations from 0 to 10 mM (specified).

#### HEDP

Cells were grown in f/2 FSW with the addition of HEDP (50 μM) (Sigma Aldrich, Poole, UK). Before the inoculation of cells, the pH of the f/2 plus HEDP medium was adjusted to pH 8.2 using 1 M NaOH and the medium was sterile filtered (0.22 μm) (PALL, Port Washington, NY, USA).

#### Ge/Si manipulation

Low‐Si seawater was collected in early summer (May 2015) from the western English Channel (station L4). This batch of seawater was used for all Ge addition experiments and [dSi] was determined to be 2.0 μM using a silicate–molybdate–ascorbate assay (Kirkwood, 1989). *Coccolithus braarudii* cultures were grown in a Ge/Si ratio of 0.2 to disrupt calcification. Ge was added in the form of GeO_2_ to a final concentration of 2 or 20 μM (specified). [dSi] was amended by the addition of Na_2_SiO_3_.5H_2_O to give a final [dSi] of 10 or 100 μM (specified). For growth experiments, coccolithophore cultures were acclimated to the appropriate [dSi] for several generations (> 2 wk) before the investigation.

#### Very low Si

As it is difficult to routinely obtain natural seawater with [dSi] < 1 μM, [dSi] was further depleted using growth of the diatom *Thalassiosira weissflogii* (PLY541), as described previously (Timmermans *et al*., 2007; Durak *et al*., 2016), termed diatom deplete seawater (DDSW). Diatoms were removed by sterile filtration and f/4 nutrients were added (without Si). [dSi] was below the level of detection (< 0.2 μM) in all DDSW media prepared by this method. Coccolithophore cultures were acclimated to DDSW for several generations (> 2 wk) before the investigation with amended [dSi] (addition of Na_2_SiO_3_·5H_2_O) to 20 μM. Before inoculation, *C. braarudii* cells were washed with < 0.2 μM [dSi] DDSW to avoid carry‐over of dSi. Cells were grown in semi‐continuous batch cultures, control and very low [dSi] (20 and < 0.2 μM, respectively) DDSW, subculturing every 9 d into fresh media to maintain cells in exponential growth.

### Measurements of photosynthesis

Measurements of chlorophyll fluorescence were taken to assess the performance of the photosynthetic apparatus. The maximum quantum yield of photosystem II (*F*_v_/*F*_m_) was determined using a Z985 AquaPen chlorophyll fluorimeter (Qubit Systems, Kingston, ON, Canada). Cells were dark adapted for 20 min before measurements. Cell densities of > 10 000 cells ml^−1^ were required to produce consistent *F*_v_/*F*_m_ measurements. O_2_ evolution measurements were performed using a Firesting O_2_ meter with an OXVIAL 4 respiration vial with integrated optical oxygen sensor (Pyro Science, Aachen, Germany). Cells were stirred constantly during measurements and kept at 20°C using a water‐cooled glass jacket. As high cell densities are required for robust O_2_ evolution measurements in *C. braarudii* (> 35 000 cells ml^−1^), cells for analysis were grown to late exponential phase in ASW at 10 mM Ca^2+^, washed and incubated in different Ca^2+^ concentrations (0, 1 and 10 mM) for 24 h before being placed in the O_2_ vial. A dark period of at least 5 min was used to record the respiration rate and then O_2_ evolution was monitored with illumination at 200 μmol photons m^−2^ s^−1^ for 5 min.

### Time‐lapse microscopy

Light microscopy images were acquired using a DMi8 Inverted Microscope with a DFC700 T colour camera (Leica Microsystems, Milton Keynes, UK). During time‐lapse imaging, cells were placed on a cooled stage at 17°C. For time‐lapse imaging of cell division, cells were maintained in the dark and illuminated only for image capture (exposure, 300 ms; frame rate, 5 min). Approximately 10–20 cells were viewed simultaneously for each time lapse. Where stated, cells were gently decalcified with 0 mM Ca^2+^ ASW at pH 7.0 for 1 h before resuspension in FSW f/2. To monitor the response to Ge treatment, cells were grown in 40 ml of culture and 1 ml aliquots were removed every 24 h for time‐lapse imaging over a period of 12 h. Cells were maintained on the microscope in constant light to encourage calcification. Approximately 100–120 cells were viewed simultaneously for these time lapses. Images and sequences were processed using Leica Applications Suite X and ImageJ (Abràmoff *et al*., 2004) software.

### Fluorescence microscopy

Nuclei of Ge‐treated cells were stained with Hoechst 33342 (Invitrogen) at a final concentration of 1 μg ml^−1^, and incubated in the dark at 15°C for 1 h. The cells were then stained with FM 1‐43 (*N*‐(3‐triethylammoniumpropyl)‐4‐(4‐(dibutylamino) styryl) pyridinium dibromide) (Thermo Fisher, Loughborough, UK) immediately before imaging with a DMi8 Inverted Microscope (Leica Microsystems) with an ORCA Flash 4.0 camera (Hamamatsu, Hamamatsu, Japan). Hoescht 33342 was excited at 395 nm with emission at 435–485 nm. FM 1‐43 was excited at 470 nm with emission at 500–550 nm. Extracellular polysaccharides were stained using the fluorescent lectin, FITC‐Concanavalin A (100 μg ml^−1^). Cells were rapidly decalcified *in situ* on the microscope to ensure that the occurrence of paired cells was not induced by the decalcification process; 1 ml of *C. braarudii* cells was decalcified following the addition of 10 μl of 1 M HCl for 10 min. pH was then restored by the addition of an equal volume of 1 M NaOH. Cells were imaged using a Zeiss LSM 510 laser scanning confocal microscope (Zeiss, Cambridge, UK), with excitation at 488 nm and emission at 500–530 nm (FITC) and 650–715 nm (chlorophyll).

### Scanning electron microscopy (SEM)

Samples for SEM were filtered onto a 13‐mm, 0.4‐μm Isopore filter (Millipore EMD, Watford, UK) and rinsed with 5 ml of 1 mM HEPES‐buffered (pH 8.2) MilliQ water to remove any salt. Filters were air dried, mounted onto an aluminium stub and sputter coated with 10 nm Pt/Pd (Cressington, Watford, UK). Samples were examined using a Phillips XL30S FEG SEM (FEI‐Phillips, Hillsboro, OR, USA) and imaged in high‐resolution secondary electron mode with a beam acceleration of 5 kV. SEM was used to score malformed, incomplete and normal coccoliths for each cell examined (> 30 cells per sample).

### Immunofluorescence microscopy

Samples were prepared for immunofluorescence microscopy as described by Durak *et al*. (2017). Briefly, *C. braarudii* cells were decalcified with Ca^2+^‐free ASW pH 8.0 containing 25 mM ethylene glycol‐bis(β‐aminoethyl ether)‐N,N,N′,N′‐tetraacetic acid (EGTA). Cells were then fixed for 10 min in an ASW solution containing 2% glutaraldehyde and 1.7% BSA (bovine serum albumin). Samples were washed three times with a solution of ASW with 1.7% BSA and 0.5% glutaraldehyde, and then incubated for 10 min in 0.05% Triton X‐100 in ASW. Samples were then washed three times with ASW/1.7% BSA and incubated for a further 20 min. Fixed samples were incubated overnight in a 1/50 dilution of the primary anti‐α‐tubulin antibody, washed three times with ASW/1% BSA and then incubated in a 1/150 dilution of the secondary Texas Red‐conjugated antibody for 2.5 h. Cells were then washed a final three times with ASW/1.7% BSA. Cells were imaged using an LSM 510 confocal laser scanning microscope (Zeiss). Texas Red was excited at 543 nm, with emission at 575–625 nm. Calcite was imaged using reflectance, with excitation at 633 nm and a short‐pass emission filter at 685 nm.

## Results

### Disruption of calcification in *C. braarudii*

**Figure 1:**
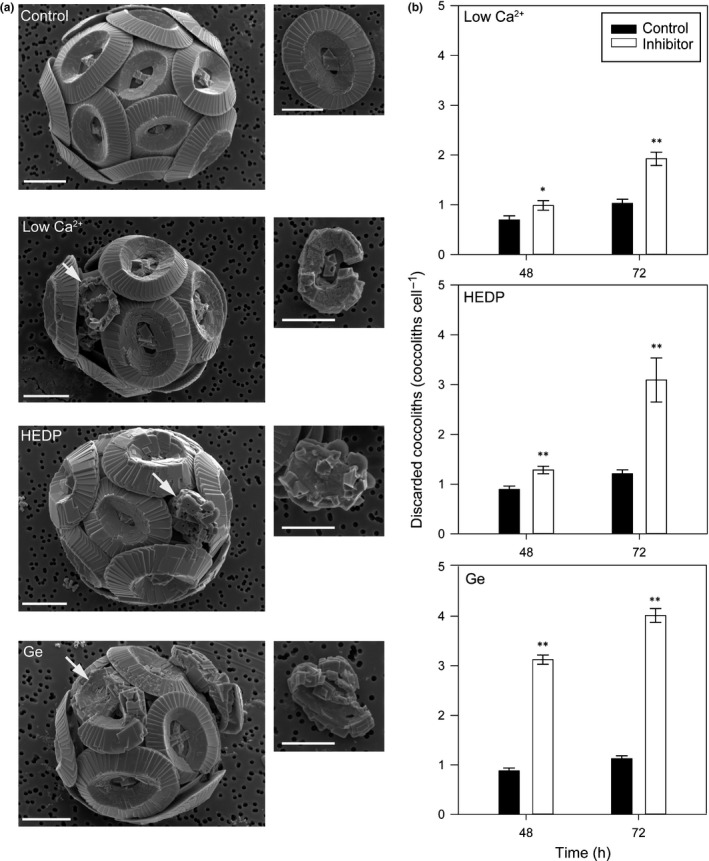
Disruption of calcification in *Coccolithus braarudii*. (a) Representative scanning electron microscopy (SEM) images of *C. braarudii* cells grown in 1 mM Ca^2+^ (48 h), 5 μM HEDP (24 h) and 0.2 germanium (Ge)/silicon (Si) (100 μM Si, 24 h). Incomplete or malformed coccoliths can be observed in response to all three treatments (arrows), whereas these are largely absent from control cells. Insets show representative incomplete or malformed coccoliths in greater detail. Incomplete coccoliths are defined as those that exhibit the oval shape of control coccoliths, but calcite precipitation is not complete. Malformed coccoliths are defined as coccoliths with gross defects in crystal morphology and no longer resemble the oval morphology of control coccoliths. Bars, 5 μm. (b) Treatments used to disrupt calcification in *C. braarudii* result in a significant increase in discarded coccoliths per cell (*, *P* < 0.05; **, *P* < 0.01, one‐way ANOVA with Holm–Sidak *post hoc* test), indicative of incomplete or malformed coccoliths that fail to integrate successfully into the coccosphere. Error bars denote ± SE. *n* = 3.

We examined the physiological effects of disrupting calcification in *E. huxleyi* and *C. braarudii* using multiple independent methodologies: low‐Ca^2+^ seawater, the addition of HEDP or the addition of Ge. Ge was not applied to *E. huxleyi,* as we have demonstrated previously that this species does not require Si for calcification and is consequently unaffected by Ge, even at very high Ge/Si ratios (Durak *et al*., 2016). Calcification was substantially disrupted in *E. huxleyi* cultures grown at 1 mM Ca^2+^ or in the presence of 50 μM HEDP, consistent with previous reports (Sekino & Shiraiwa, 1994; Herfort *et al*., 2002; Trimborn *et al*., 2007; Leonardos *et al*., 2009; Mackinder *et al*., 2011). All three treatments (low Ca^2+^, HEDP and Ge) also had profound and specific impacts on the calcification process in *C. braarudii*. SEM revealed the presence of one to two incomplete coccoliths in *C. braarudii* cells grown at 1 mM Ca^2+^ for 48 h, suggesting that this treatment interfered with the ability to form new coccoliths, but did not cause extensive dissolution of existing coccoliths (Fig. 1a; Supporting Information, Table S1). Treatment with 50 μM HEDP resulted in grossly malformed coccoliths that could be initially observed after 24 h and were abundant after 72 h. Cells exposed to Ge at a Ge/Si ratio of 0.2 generated highly malformed coccoliths within 24 h that were morphologically distinct from the malformed coccoliths formed following HEDP treatment. Polarized light microscopy of decalcified *C. braarudii* cells after 24 h of Ge treatment allowed us to confirm that the coccolith malformations occur internally, within the coccolith vesicle (Fig. S1). We have observed previously that malformed coccoliths in *C. braarudii* often fail to integrate into the coccosphere and accumulate in the seawater medium around the cell (Durak *et al*., 2016). All three treatments applied to *C. braarudii* cells in this study resulted in a significant increase in discarded coccoliths after 48 and 72 h (Fig. 1b), indicating that many of the newly produced coccoliths were not incorporated into the coccosphere. Thus, although the cells continue to calcify and produce coccoliths following treatment with low Ca^2+^, HEDP or Ge, their ability to maintain a complete coccosphere is compromised.

### Disruption of calcification inhibits growth in *C. braarudii*

**Figure 2:**
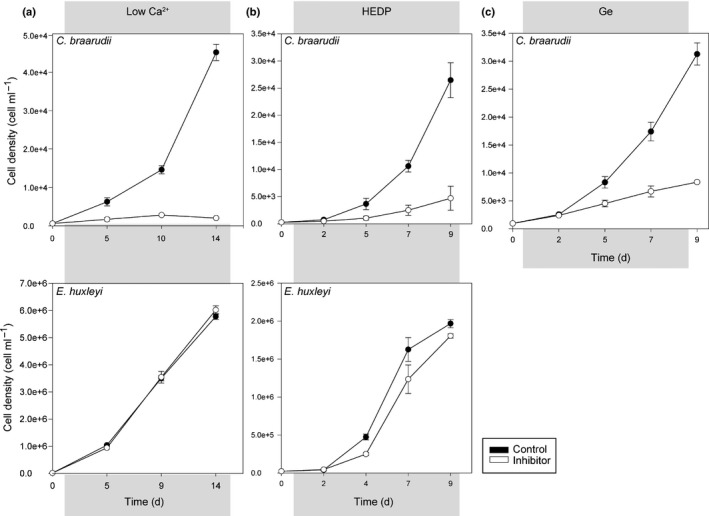
Disruption of calcification leads to a reduction in growth in *Coccolithus braarudii*. (a) Growth of *C. braarudii* and *Emiliania huxleyi* at 1 or 10 mM Ca^2+^ for 14 d. The specific growth rate (SGR ± SE) of *E. huxleyi* was not significantly different at 1 mM Ca^2+^ relative to the 10 mM Ca^2+^ control (0.55 ± 0.006 and 0.55 ± 0.002 d^−1^, respectively, *P* = 0.91, two‐tailed *t*‐test), whereas the growth of *C. braarudii* was severely inhibited at 1 mM Ca^2+^ (SGR ± SE = 0.16 ± 0.01 d^−1^) relative to the control (SGR ± SE = 0.32 ± 0.01 d^−1^, *P* < 0.05). (b) The growth of *C. braarudii* in 50 μM HEDP for 9 d was significantly reduced compared with the control (SGR ± SE = 0.30 ± 0.05 and 0.53 ± 0.01 d^−1^, respectively, *P* < 0.05), whereas the growth of *E. huxleyi* was not significantly different (SGR ± SE: 50 μM HEDP, 0.66 ± 0.03 d^−1^; control, 0.76 ± 0.08 d^−1^; *P* = 0.31). (c) The growth of *C. braarudii* in the presence of germanium (Ge; 0.2 Ge/silicon (Si)) for 9 d was significantly reduced relative to the control (SGR ± SE = 0.20 ± 0.04 d^−1^ compared with 0.38 ± 0.03 d^−1^ in the control, *P* < 0.05). Error bars denote ± SE and, in all cases, a two‐tailed *t*‐test was used. *n* = 3.

Disruption of calcification with 1 mM Ca^2+^ or 50 μM HEDP had dramatically different effects on growth in *E. huxleyi* and *C. braarudii* (Fig. 2a,b). *Emiliania huxleyi* did not exhibit any significant change in growth at 1 mM Ca^2+^ or 50 μM HEDP, confirming previous reports (Sekino & Shiraiwa, 1994; Herfort *et al*., 2002, 2004; Shiraiwa, 2003; Trimborn *et al*., 2007; Leonardos *et al*., 2009), whereas growth of *C. braarudii* was severely inhibited by both treatments. The growth of *C. braarudii* was also severely inhibited following treatment with Ge (0.2 Ge/Si) for 9 d (Fig. 2c). Thus, disruption of calcification by multiple methods has little impact on growth in *E. huxleyi*, but results in severe inhibition of growth in *C. braarudii*, suggesting that the requirement for calcification is very different between these species.

All three treatments (Ge, HEDP and low Ca^2+^) have distinct impacts on coccolith morphology, suggesting that each acts to disrupt calcification directly, rather than causing defects in coccolith morphology indirectly via reduced growth. In support of this hypothesis, the defects in coccolith morphology in response to Ge and HEDP treatment arise very rapidly (Table S1), before any defect in growth is observed (Fig. 2). The coccolith malformations are also distinct from those arising from nutrient limitation or temperature stress (Gerecht *et al*., 2014, 2015).

### Low Si inhibits growth when coccosphere formation is disrupted

**Figure 3:**
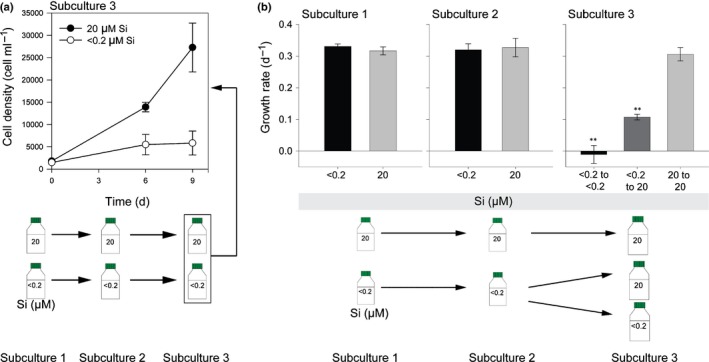
Disruption of calcification by limitation of silicon (Si) availability. (a) Growth of *Coccolithus braarudii* at < 0.2 μM [dSi] in semi‐continuous batch culture for 27 d. Cells were subcultured every 9 d. No effect of Si limitation was observed on growth in the first two subcultures (0–9 d, 9–18 d). In the third sub‐culture (18–27 d), growth at < 0.2 μM [dSi] was greatly reduced compared with cultures maintained at 20 μM [dSi] (*n* = 3). The experiment was repeated twice more with similar results. (b) Rescue of Si‐limited cultures. Cells grown in < 0.2 μM [dSi] for 21 d (subcultures 1 and 2) were transferred into media containing < 0.2 or 20 μM [dSi] (subculture 3). Growth in subculture 3 was absent at < 0.2 μM [dSi]. However, growth was partially restored in cells transferred from < 0.2 μM to 20 μM [dSi] (**, *P* < 0.01, SGR calculated 7–14 d after Si resupply, one‐way ANOVA with Holm–Sidak *post hoc* test, *n* = 3 biological replicates unnecessary as stated in methods). Error bars denote ± SE.

We have shown previously that *C. braarudii* exhibits subtle defects in coccolith morphology after 3 d in very low [dSi] (< 0.2 μM), although cells monitored for up to 8 d exhibited no decrease in growth rate (Durak *et al*., 2016). As the requirement for Si is likely to be low in *C. braarudii* (compared with silicified organisms), we grew the cells at very low [dSi] (< 0.2 μM) for longer periods (27 d, subculturing the cells every 9 d) to ensure that any intracellular pools of Si were depleted. Light microscopy observations at 9 d and 18 d did not reveal clear defects in the coccosphere at < 0.2 μM [dSi] (Fig. S2) compared with the control (20 μM [dSi]), and no effects on growth were observed (Fig. 3a). However, after transfer to the third subculture, cells at < 0.2 μM [dSi] were observed with incomplete or partial coccospheres after 21 d, whereas cells at 20 μM [dSi] were fully calcified (Fig. S2). Growth was also greatly reduced at < 0.2 μM [dSi] during the third subculture compared with the control (SGR ± SE of 0.11 ± 0.08 and 0.29 ± 0.03 d^−1^, respectively, *P* ≤ 0.05, *n* = 3, one‐tailed *t*‐test) (Fig. 3a). To test whether the inhibition of growth caused by Si limitation was reversible, we transferred poorly calcified cells grown at < 0.2 μM [dSi] for 21 d into media containing < 0.2 μM or 20 μM [dSi]. The cells transferred to < 0.2 μM [dSi] did not demonstrate any further growth after 21 d and still possessed incomplete or partial coccospheres. However, the cells transferred from < 0.2 μM [dSi] to 20 μM [dSi] exhibited fully formed coccospheres within 7 d of the resupply of Si, and growth was partially restored after this time point (Figs 3b, S2). The delayed growth response to Si addition suggests that the recovery of an Si‐dependent process, such as calcification, is responsible for the growth rescue rather than simply the resupply of Si.

### Disruption of calcification does not inhibit photosynthesis

**Figure 4:**
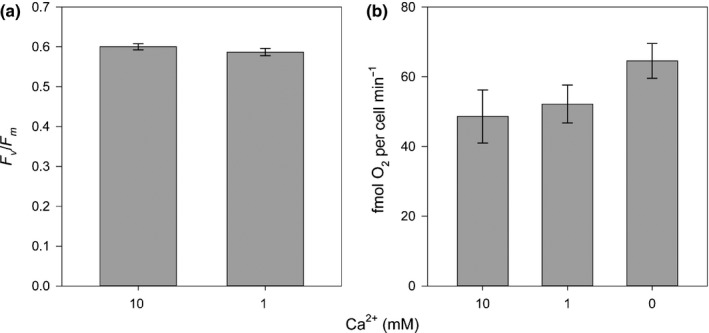
Disruption of calcification with low Ca^2+^ does not inhibit photosynthetic activity. (a) Photosynthetic efficiency (quantum yield, *F*_v_/*F*_m_) was measured in *Coccolithus braarudii* cultures incubated in artificial seawater (ASW) containing 1 or 10 mM Ca^2+^ for 72 h. No significant difference in *F*_v_/*F*_m_ was observed relative to the control (*P* ≥ 0.05, *n* = 3, two‐tailed *t*‐test). (b) Photosynthetic O_2_ evolution in *C. braarudii* cultures after growth in ASW with 0, 1 or 10 mM Ca^2+^ for 24 h. Disruption of calcification with 0 or 1 mM Ca^2+^ did not result in a statistically significant change in the rate of O_2_ evolution (*P* ≥ 0.05, *n* = 3, one‐way ANOVA). Error bars denote ± SE. The experiment was repeated twice; a representative example is shown.

We examined whether inhibition of growth following disruption of calcification was caused by an effect of calcification on photosynthesis, such as acting as a carbon‐concentrating mechanism or modulating light entry into the cell. Disruption of calcification with low Ca^2+^ (1 mM), 50 μM HEDP or 20 μM Ge (0.2 Ge/Si ratio) had no impact on the photosynthetic efficiency of photosystem II (quantum yield, *F*_v_/*F*_m_) in *C. braarudii* cells after 72 h of treatment, suggesting that the absence of calcification did not lead to broad disruption of the photosynthetic apparatus (Figs 4a, S3). Similarly, we observed no decrease in the rate of photosynthetic O_2_ evolution in cells transferred to 0 or 1 mM Ca^2+^ for 24 h relative to the control (10 mM Ca^2+^) (one‐way ANOVA, *P* = 0.90, *n* = 3) (Fig. 4b). We conclude that direct inhibition of photosynthetic function does not appear to be responsible for the reduction in growth in *C. braarudii* following disruption of calcification. Moreover, the absence of a significant effect on photosynthetic efficiency after 72 h indicated that the treatments used to disrupt calcification do not lead to the disruption of general cellular function.

### The role of the coccosphere during cell division

**Figure 5:**
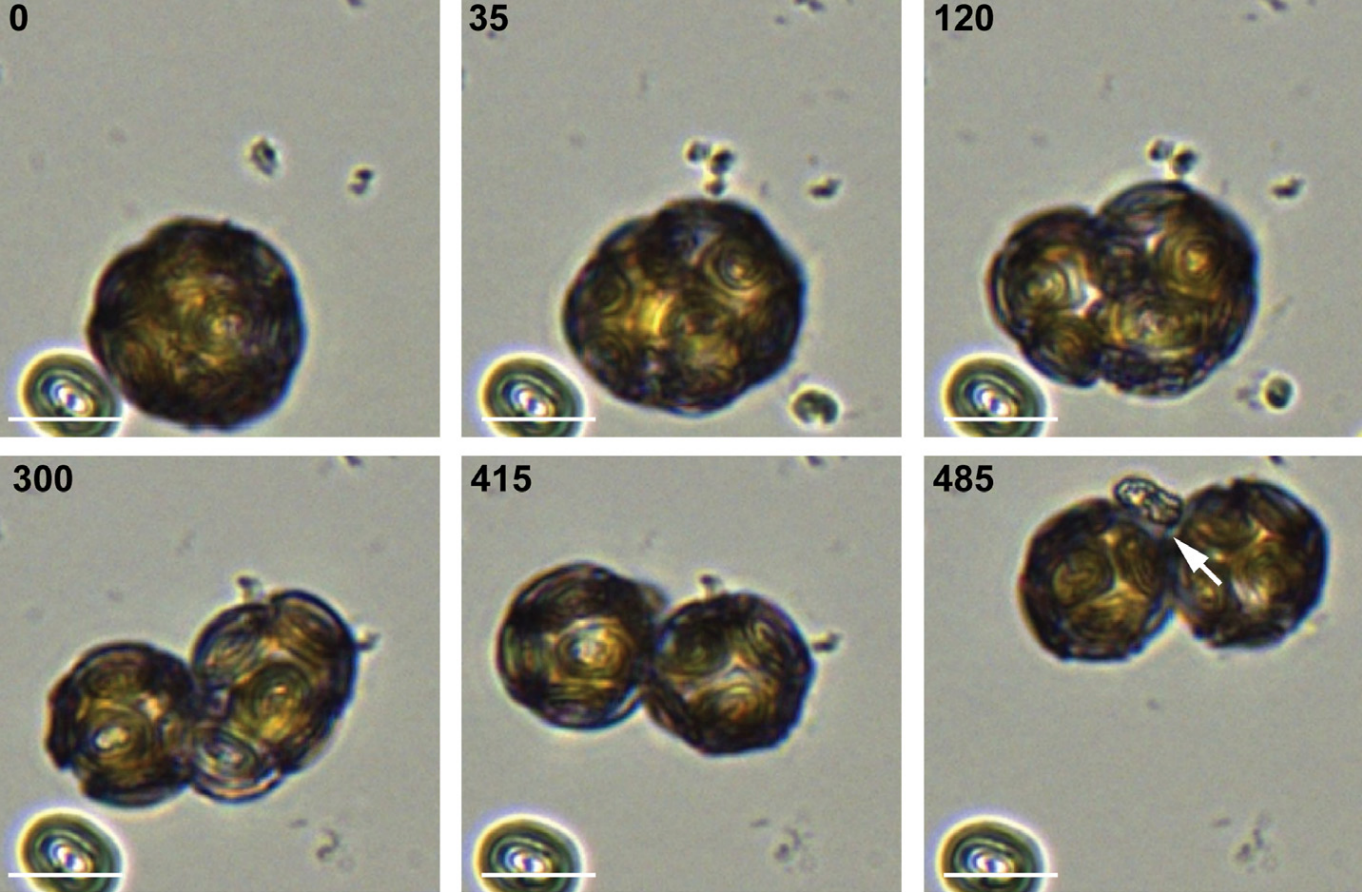
Rearrangement of the coccosphere during cell division. Time‐lapse light microscopy imaging of *Coccolithus braarudii* undergoing cell division recorded over 16 h in the dark (16°C). At the onset of cell division, the cell begins to elongate and the coccoliths move flexibly on the cell surface to maintain a complete coccosphere (35 min). As the cell divides (300 min), the coccosphere rearranges to ensure that both daughter cells are fully covered following division (415 min). In the example shown, a partially formed coccolith is secreted during division (arrowed), implying that cell division occurs regardless of whether coccolith production is completed. Bars, 10 μm.

We next investigated whether the inhibition of growth resulted from the inability of *C. braarudii* to form a complete coccosphere. Removal of the coccosphere does not lead to an immediate loss of cell viability in *C. braarudii* as decalcified cells continue to calcify and eventually form a complete new coccosphere (Taylor *et al*., 2007, 2017). However, the mechanisms enabling the reorganization of the coccosphere during cell division are not known, and it is possible that disruption of calcification interferes with this process. Coccolithophore cells become larger during the day and, once they surpass a size threshold (Müller *et al*., 2008), divide into two smaller daughter cells during the dark period. Although there have been some previous observations of cell division using light microscopy (Parke & Adams, 1960), direct visualization of the process in live cells has not been reported. Using time‐lapse imaging, we found that dividing *C. braarudii* cells elongate immediately before cell division (Fig. 5; Video S1). The coccoliths move flexibly to span the fissure between the two daughter cells before closing in a hinge‐like motion to form two distinct, but attached, cells. The coccoliths undergo further rearrangement and, once both daughter cells have complete coccospheres, the cells separate. The remarkable flexibility in the coccosphere ensures that *C. braarudii* is able to rearrange its closely interlocking coccoliths to cover the dividing cell throughout the entire process. We observed that cells remained attached for a short period after division, but then separated between 4 and 7 h later (*n* = 4 cells undergoing both division and separation within a 12‐h time course; Fig. S4). Interestingly, secretion of a partially formed or complete coccolith was observed during the process of cell division (Fig. 5) (38.1% of all division events observed, *n* = 21). This suggests that the intracellular coccolith may interfere with the rearrangement of the cytoskeleton during cell division and is therefore exocytosed, even if it is only partially formed, which is consistent with previous light microscopy observations noting the absence of an internal coccolith in dividing cells (Parke & Adams, 1960).

**Figure 6:**
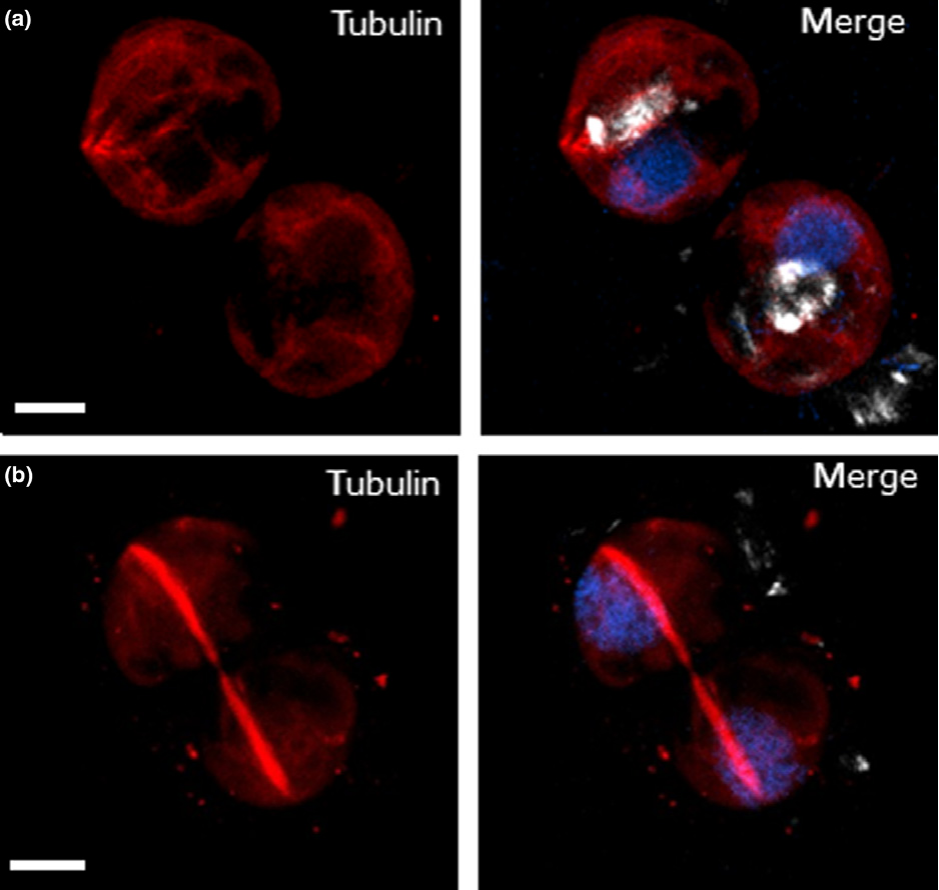
Immunofluorescence microscopy of tubulin in dividing *Coccolithus braarudii* cells. Cells were decalcified before imaging. (a) Three‐dimensional (3D) projection of a confocal microscopy *Z*‐stack showing the presence of internal coccoliths in non‐dividing cells (white). The nuclei are stained with Hoescht (blue) and tubulin is shown in red. Note that there is some non‐specific background fluorescence in the red channel caused by fixation with glutaraldehyde. (b) The microtubule network in dividing *C. braarudii* cells is characterized by a distinct microtubule bundle that spans both daughter cells. Two distinct nuclei can be observed, but intracellular calcite is absent. Image is representative of 14 cells examined. Bars, 5 μm.

To examine the interaction between calcification and cell division in more detail, we used immunofluorescence microscopy to image the microtubule network during cell division. In dividing cells, a very clear microtubule bundle can be observed which spans both cells, persisting even after full separation of the daughter nuclei (Fig. 6). Intracellular coccoliths are present in nearly all non‐dividing cells (85.3% of cells exhibit distinct coccoliths and a further 11.8% exhibit smaller accumulations of intracellular calcite, *n* = 68 cells), whereas coccoliths are absent from dividing cells (*n* = 14). These data illustrate the requirement for significant rearrangement of the cytoskeleton during cell division in coccolithophores. The absence of internal coccoliths from dividing cells supports our observation that coccoliths are secreted before cell division.

### Disruption of the coccosphere prevents separation following cell division

**Figure 7:**
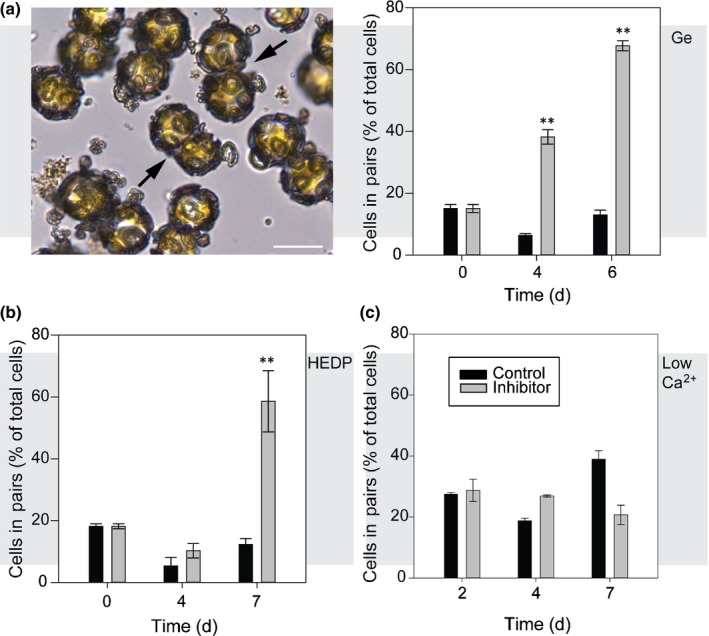
Paired cells accumulate in cells with disrupted calcification. (a) Paired cells (arrowed) accumulate in germanium (Ge)‐treated *Coccolithus braarudii* repetitive of word cells (2 μM Ge, 0.2 Ge/silicon (Si)). The graph shows the percentage of cells present as pairs (viewed by light microscopy). *n* > 100 cells for each measurement. Bar, 20 μm. (b) Percentage of cells present as pairs in *C. braarudii* cells treated with 50 μM HEDP. (c) Percentage of cells present as pairs in *C. braarudii* cells grown in artificial seawater (ASW) at 1 mM Ca^2+^, relative to control cells at 10 mM Ca^2+^. No increase in cells in pairs was observed in the low‐Ca^2+^ treatment. **, *P* < 0.01, one‐tailed *t*‐test. *n* = 3 replicates for treatments. Error bars denote ± SE.

To test whether an intact coccosphere was required for entry into the cell cycle, decalcified *C. braarudii* cells were observed by time‐lapse microscopy for 12 h. We observed that fully decalcified cells undergo cytokinesis, indicating that the absence of a coccosphere does not prevent entry into and progression through the cell cycle (Fig. S5). However, closer inspection of HEDP‐ and Ge‐treated cells revealed that many cells are present in pairs, comprising two cells closely attached to each other (Fig. 7). The number of paired cells increased progressively following treatment, with up to 68% of cells present as pairs after treatment with Ge (0.2 Ge/Si) or 50 μM HEDP for 6 or 7 d, respectively (Fig. 7a,b). The paired‐cell phenotype was not apparent in cells grown at 1 mM Ca^2+^, suggesting that the mechanism of growth inhibition may differ in low Ca^2+^ (Fig. 7c).

**Figure 8:**
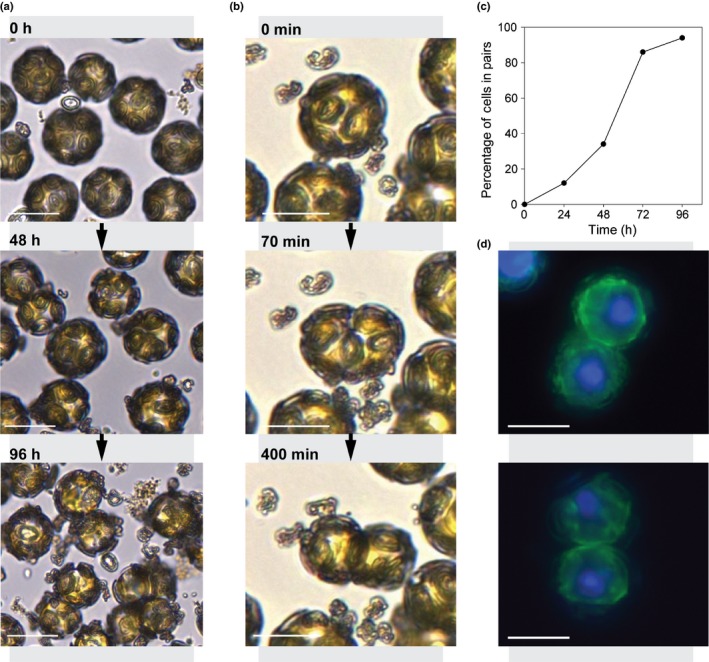
Progressive disruption of the coccosphere in *Coccolithus braarudii* cells treated with germanium (Ge). (a) Time‐lapse light microscopy showing the progressive degradation of the coccosphere and the accumulation of paired cells in *C. braarudii* cells treated with 2 μM Ge (0.2 Ge/silicon (Si)) over a 96‐h period. Cells exhibit intact coccospheres at 0 h, but start to produce malformed coccoliths soon after the addition of Ge. After 96 h, most cells exhibit incomplete coccospheres and many are present as paired cells. (b) Time‐lapse light microscopy showing the formation of a cell pair after 3 d of Ge treatment (0.2 Ge/Si). Parent cells with partial coccospheres divide, but the daughter cells fail to fully separate. Frame labels represent minutes passed. (c) The percentage of paired cells after treatment with 2 μM Ge (0.2 Ge/Si) over 5 d (*n* ≥ 500 cells counted). (d) Epifluorescence microscopy of paired *C. braarudii* cells. The nuclei were stained with Hoechst (blue) and the plasma membrane was stained with FM 1‐43 (green). Cells were not decalcified before imaging. Each paired cell examined showed completed cytokinesis with two defined nuclei and a distinct plasma membrane. Bars, 20 μm.

Although flow cytometry is commonly used to measure cell cycle progression in unicellular organisms, we found that the fragile *C. braarudii* cells were not amenable to this approach. Furthermore, flow cytometry cannot adequately distinguish between two cells that remain attached to each other and a cell in G2/M phase. We therefore used time‐lapse microscopy to enable the direct observation of cell division, coccolith production and calcification status of individual Ge‐treated cells. Importantly, this also allowed us to obtain detailed information on the status of the coccosphere in individual cells before division. A culture of *C. braarudii* cells treated with Ge (0.2 Ge/Si) was sampled every 24 h over a period of 5 d to generate a series of individual 12‐h time‐lapse recordings. These images revealed that the initial secretion of malformed coccoliths occurs within 6 h of Ge treatment, suggesting that Ge has a rapid impact on coccolithogenesis (Figs 8a, S6). The continued production of malformed coccoliths could be observed on successive days, leading to a progressive decrease in the integrity of the coccosphere, with most cells possessing severely defective coccospheres after 5 d (Figs 8a, S7). Time‐lapse observation of individual cells indicated that paired cells form when cells divide, but fail to separate (Fig. 8b). Examination of each paired cell (*n* > 500 paired cells examined) indicated that, in every case, both daughter cells exhibited significant defects in coccosphere integrity. The number of cells exhibiting the paired‐cell phenotype increased dramatically over the course of the experiment, from 4% after 24 h, through to 89.5% after 96 h (Fig. 8c). The increasing proportion of cells present as pairs therefore correlates with both the decrease in the integrity of the coccosphere and the decrease in growth rate in Ge‐treated cells (Fig. 2).

DNA staining showed that the paired cells represented two individual daughter cells, each with a single nucleus and a distinct plasma membrane (Fig. 8d). No clear difference in DNA content was observed between control and Ge‐treated cells. We did not observe any further rounds of cell division in paired cells in time‐lapse images (i.e. leading to the formation of tetrad cell arrangements). This indicates that there is a cell cycle arrest following the initial division, which is most likely the underlying cause of growth inhibition.

### A polysaccharide‐rich organic layer contributes to cell adhesion in the absence of the coccosphere

**Figure 9:**
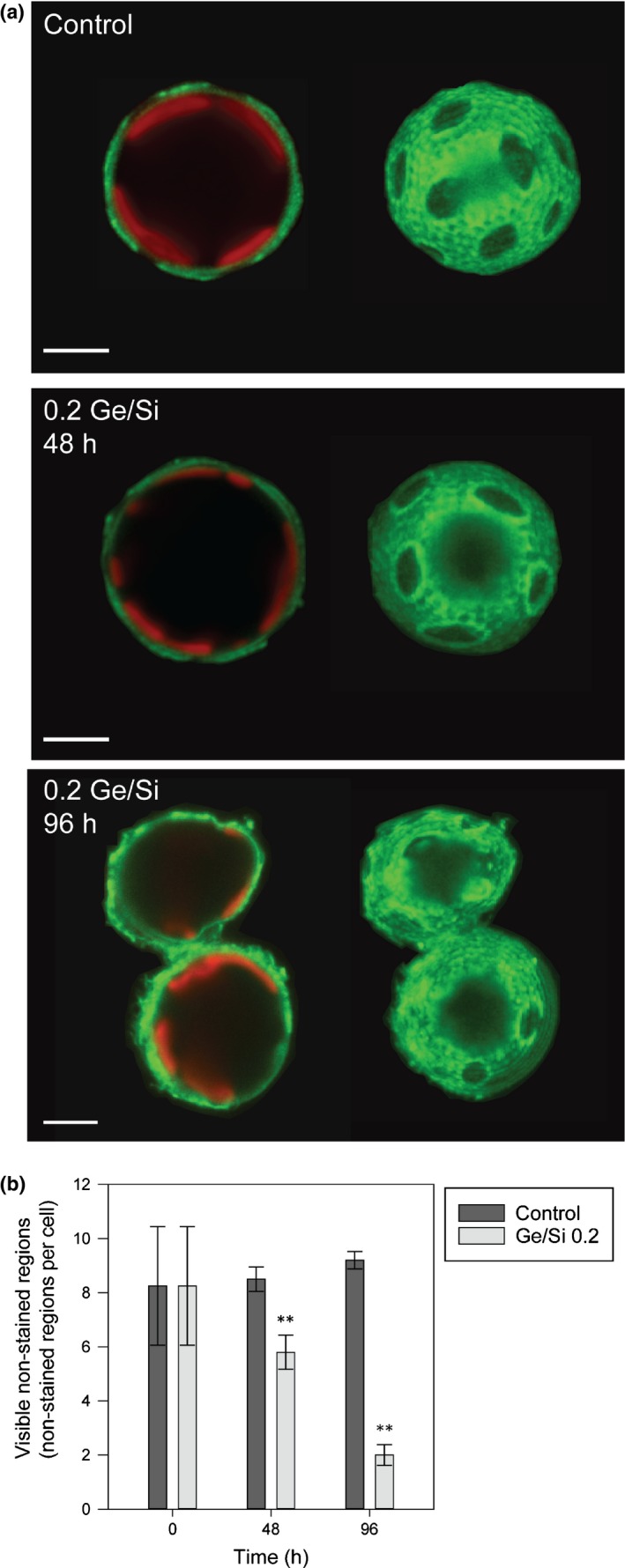
A structured polysaccharide layer is involved in organization of the coccosphere. (a) Confocal microscopy imaging of a decalcified *Coccolithus braarudii* cell stained with the lectin FITC‐Concanavalin A (green). Chlorophyll autofluorescence is also shown (red). An external polysaccharide layer can be observed that is distinct from the faint staining present at the plasma membrane (left). Three‐dimensional (3D) reconstruction from *Z*‐stacks reveals that the polysaccharide layer contains distinct non‐stained oval‐shaped regions, which are likely to correspond to the position of the coccoliths. 3D reconstructions of the cells in 0.2 germanium (Ge)/silicon (Si) (10 μM Si) at 48 and 96 h show a reduction in the number of non‐stained oval‐shaped regions per cell and increasing irregularity in their shape. Examination of paired cells present after 96 h revealed that each cell in a pair is surrounded by a continuous polysaccharide layer (FITC‐Concanavalin A, green) with staining clearly visible at the connection point between the two cells. Paired cells were first identified by light microscopy and then decalcified *in situ* to ensure that adhesion between cells was not a result of the decalcification process. Bars, 5 μm. (b) The number of visible non‐stained regions per cell was scored at 0, 48 and 96 h. There was a significant reduction (**) in visible non‐stained regions in Ge‐treated cells when compared with the control at 48 and 96 h (Mann–Whitney *U*‐test, *P* ≤ 0.01, *n* = 20). Error bars denote ± SE.

Transmission electron microscopy indicates that *C. braarudii* possesses an organic layer around the cell, which probably aids in the organization of the coccosphere and its adhesion to the cell body (Taylor *et al*., 2007). We used confocal microscopy of the fluorescent lectin, FITC‐Concanavalin A, to visualize polysaccharides in this organic layer around decalcified *C. braarudii* cells. Three‐dimensional (3D) reconstruction of the polysaccharide layer from control cells (which have an intact coccosphere before decalcification) revealed that its structure was not uniform, with distinct oval‐shaped regions present at regular intervals that were not stained by FITC‐Concanavalin A (Fig. 9a). The mean maximal diameter ± SE of the non‐stained regions was 4.22 ± 0.16 μm (*n* = 15), which is similar to the inner diameter of the shield elements of the coccolith, suggesting that these regions may correspond to apertures in the polysaccharide layer that form around each coccolith. The distinct structural properties of the polysaccharide layer, which are retained even after decalcification, are likely to contribute to the dynamic reorganization of the coccosphere throughout the processes of cell expansion and division.

*In situ* decalcification of paired cells from a Ge‐treated culture (after 96 h) revealed that each cell was surrounded by a distinct polysaccharide layer, further confirming that the paired cells are two individual cells (Fig. 9a). Direct contact between the polysaccharide layers surrounding each cell suggests that the polysaccharide contributes to cell–cell adhesion. The polysaccharide layer was more irregular and the number of non‐stained regions associated with the coccoliths was significantly decreased in Ge‐treated cells when compared with the control over 48 and 96 h (Fig. 9b, Mann–Whitney *U*‐test, *P* ≤ 0.001, *n* = 20). As Ge‐treated cells have partially formed or incomplete coccospheres at 96 h because of the inability of malformed coccoliths to integrate into the coccosphere, the data support the hypothesis that the non‐stained regions of the polysaccharide layer are apertures that are formed by the insertion of the coccoliths. We conclude that the absence of an intact coccosphere in Ge‐treated cells interferes with normal separation of dividing cells and results in cell–cell adhesion via the polysaccharide layer.

## Discussion

Our results show that disruption of calcification has dramatically different impacts on the physiology of *C. braarudii* and *E. huxleyi*. Growth of *C. braarudii* was severely inhibited following disruption of calcification by Ge, low Si, HEDP and low Ca^2+^, whereas *E. huxleyi* grew normally when calcification was disrupted by the last two treatments. Although it is possible that Ge or HEDP may have additional impacts on the metabolism of *C. braarudii*, these treatments are not generally toxic to haptophytes, as concentrations much higher than those required to disrupt calcification have little impact on the growth of *E. huxleyi* and *C. carterae* (Sekino & Shiraiwa, 1994; Asahina & Okazaki, 2004; Durak *et al*., 2016). Similarly, although Ca^2+^ is essential for many cellular processes, the lowering of seawater Ca^2+^ to 1 mM does not severely inhibit the growth of other marine phytoplankton (Herfort *et al*., 2004; Trimborn *et al*., 2007; Leonardos *et al*., 2009; Müller *et al*., 2015). Furthermore, the impact of low Si on growth of *C. braarudii* at < 0.1 μM Si was only observed following disruption of the coccosphere, suggesting that the effect on growth was specific to the defect in calcification. The combined evidence from these four independent methodologies suggests that there is an essential requirement for calcification in *C. braarudii*, but not *E. huxleyi*.

Our data highlight the dynamic nature of the coccosphere in *C. braarudii* and demonstrate the need for coordination between calcification and the cell cycle. Calcification and cell division in coccolithophores are, to some extent, temporally separated, with cell division occurring primarily in the dark, whereas calcification is largely limited to G1 phase in the light (Paasche, 2001). Our time‐lapse observations of dividing *C. braarudii* cells illustrate the rearrangement of the coccosphere during this process and the need for flexible organization of the coccosphere as the cells grow and expand between divisions. *Coccolithus braarudii* cells possess ≤ 8 coccoliths immediately after cell division, but this increases to ≥ 16 coccoliths in cells that are ready for division (Gibbs *et al*., 2013). The coccosphere of *C. braarudii* therefore represents a highly dynamic single layer of interlocking coccoliths that is maintained throughout changes in cell volume and the process of cell division. It appears that the polysaccharide layer surrounding the cell (Taylor *et al*., 2007) contributes to the organization of the coccosphere. This layer is not a simple gelatinous mass, but has sufficient structural rigidity (evidenced by the retention of coccolith‐related features in this layer following decalcification) to enable the specific arrangement of the coccoliths. However, the rapid rearrangement of the coccosphere during cell division also indicates that coccoliths are able to move within the polysaccharide layer relative to each other and that their position is not rigidly fixed.

Our experiments also provide insight into the cellular mechanisms through which disruption of calcification may act to inhibit growth in *C. braarudii*. In Ge‐ and HEDP‐treated cells, we found that the adhesive properties of the organic layer probably prevent cells with disrupted coccospheres from separating after cell division. Paired cells were also observed in Si‐limited cells with disrupted coccospheres. As paired cells fail to divide further, they may be prevented from reaching a critical size that is required for entry into S phase, leading to cell cycle arrest. Entry into S phase of the cell cycle in *E. huxleyi* is triggered by the increase in cell size above a certain threshold (Müller *et al*., 2008). Under conditions in which cells can calcify normally and maintain a complete coccosphere, the area of direct contact between dividing cells would be minimal, preventing adhesion between dividing cells. Thus, the defect in growth in cells treated with Ge, HEDP or low Si appears to result primarily from the inability to maintain a coccosphere following disruption of calcification. We did not find any evidence for a direct cell cycle arrest in Si‐limited cells analogous to that seen in diatoms (Vaulot *et al*., 1987; Brzezinski *et al*., 1990). Si limitation takes much longer than treatment with Ge to disrupt calcification. We presume that coccolithophores have a low requirement for Si, and it takes many generations for the intracellular pool of Si to become fully depleted. The rapid impact of Ge treatment (compared with Si limitation) suggests that Ge does not simply act as a competitive inhibitor of Si uptake, but also acts to disrupt the intracellular role of Si, as observed in diatoms and choanoflagellates (Azam & Volcani, 1981; Marron *et al*., 2016).

*Coccolithus braarudii* cells grown at 1 mM Ca^2+^ did not exhibit a paired‐cell phenotype, indicating that the growth arrest from this treatment did not arise from cell adhesion following disruption of the coccosphere. Although other marine phytoplankton are able to grow at 1 mM Ca^2+^ (Müller *et al*., 2015), it is possible that, in *C. braarudii*, the huge demand for Ca^2+^ in calcification leads to a broad disruption of cellular Ca^2+^ homeostasis that interferes with Ca^2+^‐dependent processes required for growth and cell division. Evidence in support of this hypothesis comes from studies in *Chrysotila* (formerly *Pleurochrysis*) *haptonemofera*, which have demonstrated that the growth of calcifying cells is inhibited at 0.5 mM Ca^2+^, whereas non‐calcifying cells grow normally at this concentration (Katagiri *et al*., 2010). Low Ca^2+^ does not disrupt growth in calcifying *E. huxleyi* cells, which may be a reflection of its ability to greatly vary rates of coccolith production (Paasche, 1998). The mechanisms of Ca^2+^ uptake and partitioning in *E. huxleyi* may also differ from those in other coccolithophores (Sviben *et al*., 2016; Gal *et al*., 2017). The absence of a paired‐cell phenotype in *C. braarudii* in low Ca^2+^ may also relate to the influence of low external Ca^2+^ on the physical properties of the extracellular polysaccharides, as many algal polysaccharides, such as pectins and alginates, are cross‐linked by Ca^2+^ and exhibit vastly different properties at lower Ca^2+^ concentrations (Corpe, 1964; Haug, 1976; Matoh & Kobayashi, 1998; Domozych *et al*., 2014).

The differing requirement to maintain a coccosphere between *C. braarudii* and *E. huxleyi* suggests that these species exhibit further mechanistic differences in the calcification process. This may relate to the different organization of the coccosphere in the two species, as the assembly of the coccosphere in *E. huxleyi* is less structured and can consist of multiple layers of coccoliths (Paasche, 2001). The coccosphere represents a uniform barrier that may help to protect the cell against external influences, such as excessive light levels, grazing by bacteria and zooplankton, or infection from pathogens. Monteiro *et al*. (2016) proposed that the requirement to protect the cell from grazing pressure may even have driven the evolution of calcification in coccolithophores *c*. 250 myr ago. In *C. braarudii*, selective pressure to maintain the coccosphere appears to have resulted in an inability to grow when calcification is disrupted. We found no evidence to suggest that the inhibition of growth in *C. braarudii* was related to impaired photosynthetic function, supporting conclusions from *E. huxleyi* that calcification does not act primarily to support photosynthesis in coccolithophores under standard laboratory conditions (Bach *et al*., 2013).

To examine whether the requirement to maintain the coccosphere may be widespread amongst other species, we performed a survey of the coccolithophore species held in major algal culture collections (Table S2). Only two lineages demonstrate the ability to routinely grow in a non‐calcified form in the diploid stage of the life cycle. The first of these groups contains solely *E. huxleyi*, whose ability to grow without coccoliths is well documented (Klaveness, 1972; Paasche, 2001). Interestingly, there are no reports that the closely related species *Gephyrocapsa* and *Reticulofenestra* are able to grow in a non‐calcified state, although all of these coccolithophores within the Noelaerhabdaceae are closely related to *Isochrysis,* which has completely lost the ability to calcify. The second group is composed of species from the Pleurochrysidaceae (*Chrysotila*) and Hymenomonadaceae (*Ochrosphaera*,* Hymenomonas*) in which the coccosphere is composed of many small coccoliths (Marsh & Dickinson, 1997; Marsh, 2004). All other coccolithophore species are fully calcified in healthy, actively growing diploid cultures. This finding suggests that the maintenance of the coccosphere in the diploid life cycle stage is a requirement for growth in many coccolithophore species, and that commonly used model organisms in laboratory studies, such as *E. huxleyi* and *C. carterae*, are not typical of coccolithophores as a whole. Many species of coccolithophore produce small holococcoliths in their haploid life cycle stage, which are distinct from the much larger heterococcoliths produced by the diploid. Intriguingly, the coccolithophore species that do not produce holococcoliths are also the species that can exist as non‐calcified diploids (e.g. *Emiliania*,* Chrysotila*,* Hymenomonas*) (De Vargas *et al*., 2007). Although it is not clear whether shared cellular mechanisms contribute to the formation of hetero‐ and holococcoliths, it is interesting that these species exhibit a lower requirement for calcification in both life cycle stages.

The essential requirement for an intact coccosphere in species such as *C. braarudii* could potentially influence their ecology and their response to future changes in ocean carbonate chemistry. The data presented here indicate that subtle impacts on calcification (such as those induced by low Si) may result in a progressive decline in the integrity of the coccosphere, eventually resulting in the inhibition of growth. A significant increase in seawater CO_2_ (*p*CO_2_ > 1000 μatm) results in a substantial decrease in both growth rate and calcification rate in *C. braarudii*, and also leads to the production of malformed coccoliths (Langer *et al*., 2006; Müller *et al*., 2010; Krug *et al*., 2011; Bach *et al*., 2015). It is interesting that prolonged growth of *C. braarudii* at elevated CO_2_ (> 45 d) resulted in a progressive decline in growth rate (Müller *et al*., 2010). Clearly, the responses of coccolithophores to changes in seawater carbonate chemistry are complex and involve many aspects of cellular physiology, but it is possible that accumulated defects in coccolith morphology and a resultant decline in coccosphere integrity could directly contribute to high CO_2_‐related growth defects in *C. braarudii*. This is an important consideration, as it reflects a potential direct impact of decreased calcification on physiology, which is not observed for *E. huxleyi*.

In summary, our results show that the ability of diploid *E. huxleyi* cells to persist in a non‐calcifying form is not typical of all coccolithophores. The requirement for calcification in *C. braarudii* is primarily a result of its need to maintain a full coccosphere, indicating that it is the coccosphere, rather than simply the ability to precipitate calcite, that is central to its ecology.

## Author contributions

C.E.W. performed all the experimental analyses, except immunofluorescence imaging of cell division, which was provided by G.M.D. S.H. provided additional imaging of extracellular polysaccharides. G.L. and A.R.T. additionally contributed to SEM analysis. I.P. provided details of coccolithophore strains in the Roscoff Culture Collection. G.L.W., C.B. and C.E.W. designed the study. C.E.W., G.L., T.T., A.R.T., C.B. and G.L.W. wrote the manuscript.

## Supporting information

Please note: Wiley Blackwell are not responsible for the content or functionality of any Supporting Information supplied by the authors. Any queries (other than missing material) should be directed to the *New Phytologist* Central Office.

**Fig. S1** Images of internal malformed coccoliths.
**Fig. S2** Images of silicon (Si)‐depleted cultures.
**Fig. S3** Photosynthetic efficiency following disruption of calcification.
**Fig. S4** Time‐lapse microscopy of cell division in *Coccolithus braarudii*.
**Fig. S5** Cell division can occur in the absence of a coccosphere.
**Fig. S6** Malformed coccolith production in germanium (Ge)‐treated cells.
**Fig. S7** Germanium (Ge)‐treated cells exhibit a progressive disruption of the coccosphere as the cell volume increases.
**Table S1** Disruption of calcification in *Coccolithus braarudii* by low Ca^2+^, HEDP or Ge, determined as the percentage of incomplete or malformed coccoliths in the coccosphere
**Table S2** The calcification status of diploid coccolithophore strains in algal culture collections

**Video S1** Cell division in *Coccolithus braarudii*.
