# Supplementary material for: The requirement for calcification differs between ecologically important coccolithophore species
Source: New Phytol. 2018 Jun 19;220(1):147–62. doi: 10.1111/nph.15272 (PMC6175242; doi:10.1111/nph.15272)
Supplement: Supplementary file 1 — Fig. S1 Images of internal malformed coccoliths. Fig. S2 Images of silicon (Si)‐depleted cultures. Fig. S3 Photosynthetic efficiency following disruption of calcification. Fig. S4 Time‐lapse microscopy of cell division in Coccolithus braarudii. Fig. S5 Cell division can occur in the absence of a coccosphere. Fig. S6 Malformed coccolith production in germanium (Ge)‐treated cells. Fig. S7 Germanium (Ge)‐treated cells exhibit a progressive disruption of the coccosphere as the cell volume increases. Table S1 Disruption of calcification in Coccolithus braarudii by low Ca2+, HEDP or Ge, determined as the percentage of incomplete or malformed coccoliths in the coccosphere Table S2 The calcification status of diploid coccolithophore strains in algal culture collections [file NPH-220-147-s001.pdf]

The requirement for calcification differs between ecologically important coccolithophore species

Charlotte E. Walker, Alison R. Taylor, Gerald Langer, Grażyna M. Durak, Sarah Heath, Ian Probert, Toby Tyrrell, Colin Brownlee, Glen L.Wheeler

Article acceptance date: 7 May 2018

Figs S1-S7

Tables S1-S2

Supplementary References

Video S1

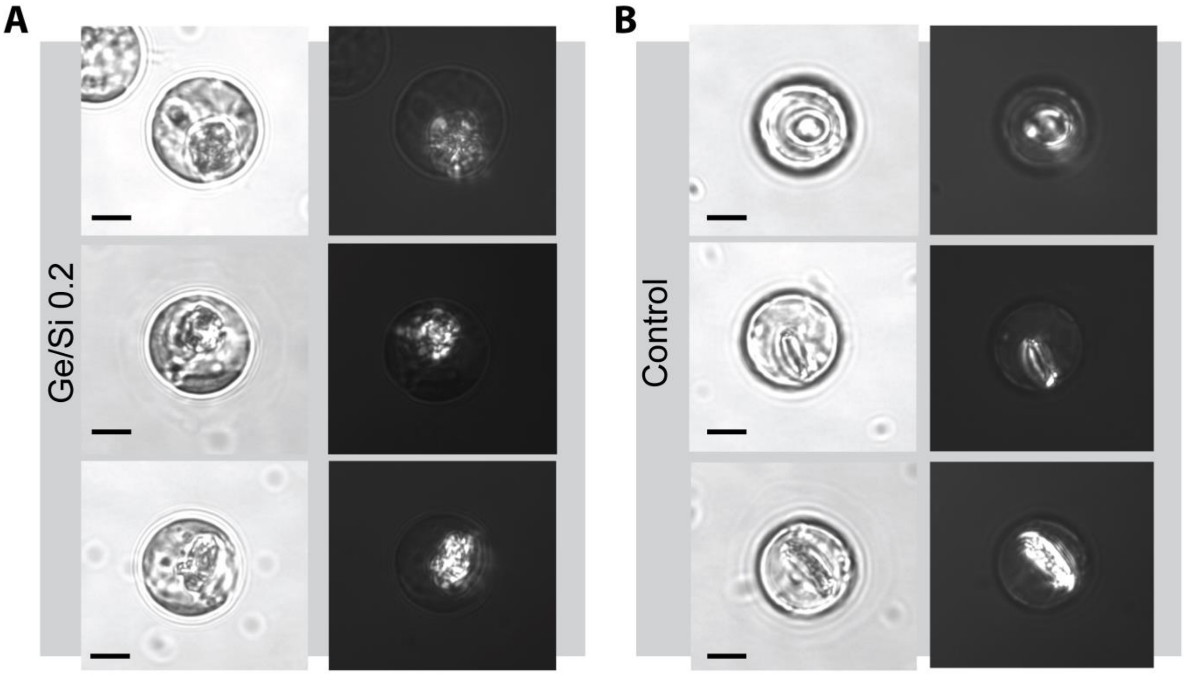

Fig. S1: Images of internal malformed coccoliths

Bright field and polarised light microscopy of decalcified *C. braarudii* cells after 24 h in 0.2 Ge/Si (A) and 0 Ge/Si control (B). Cells were decalcified prior to imaging to clearly visualise the developing internal coccolith. In the Ge treated cells coccoliths are unmistakably malformed. Ge resultant malformations are visible in both the light and polarised light images, especially when compared to the ellipsoidal structure of the control cell internal coccoliths. Scale bars denote 5 µm.

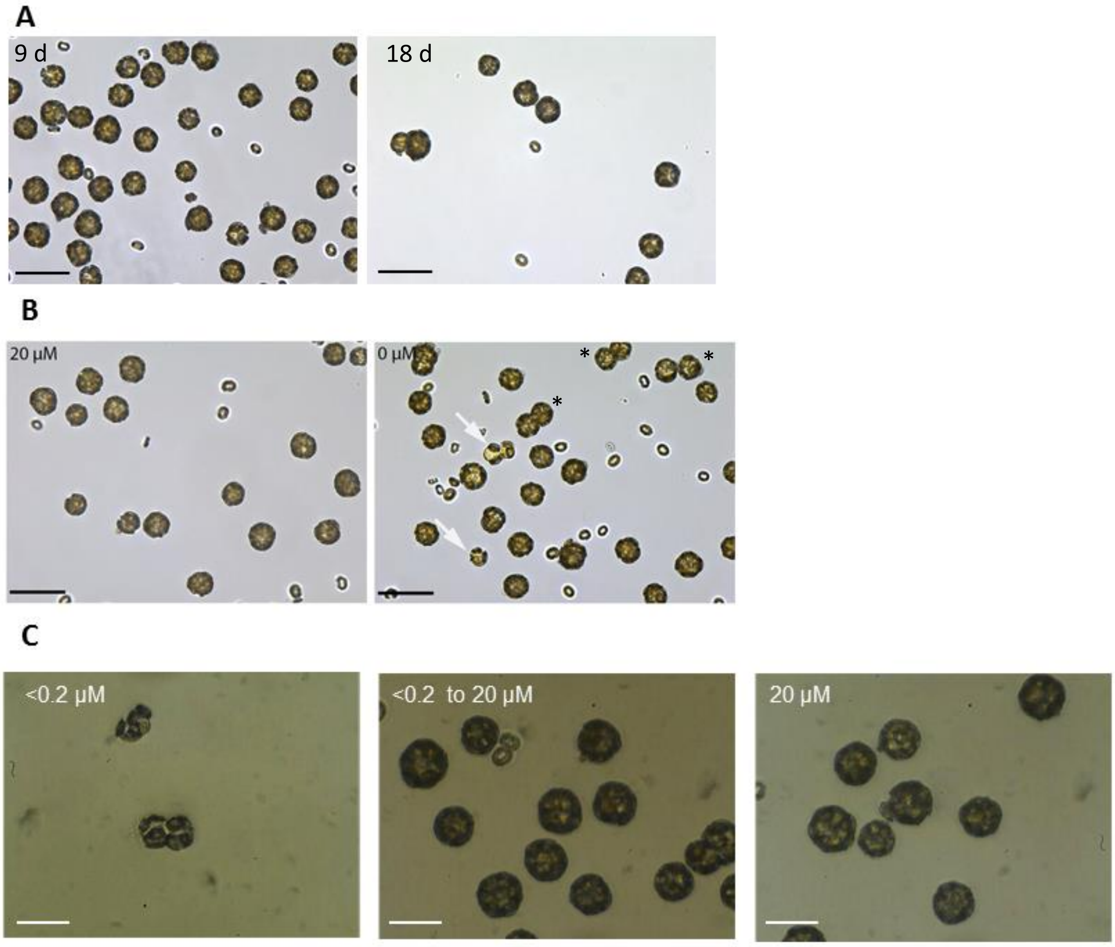

Fig. S2: Images of Si-depleted cultures

C. braarudii cells after 9 and 18 d and 21 d (third sub-culture) in <0.2 µM [dSi]. A) Cells are fully calcified at 9 and 18 d. B) Partially calcified cells can be observed at <0.2 µM [dSi] after 21 d (arrowed). Many cells are present in pairs (asterisks). C) Bright field images of cells grown in <0.2 µM [dSi] for 21 d and then transferred into <0.2 and 20 µM [dSi]. 7 d after transfer (i.e. 28 d after the initiation of the experiment) cells in <0.2 µM [dSi] are poorly calcified whereas those transferred to 20 µM [dSi] exhibit full coccospheres. Scale bars denote 50 µm.

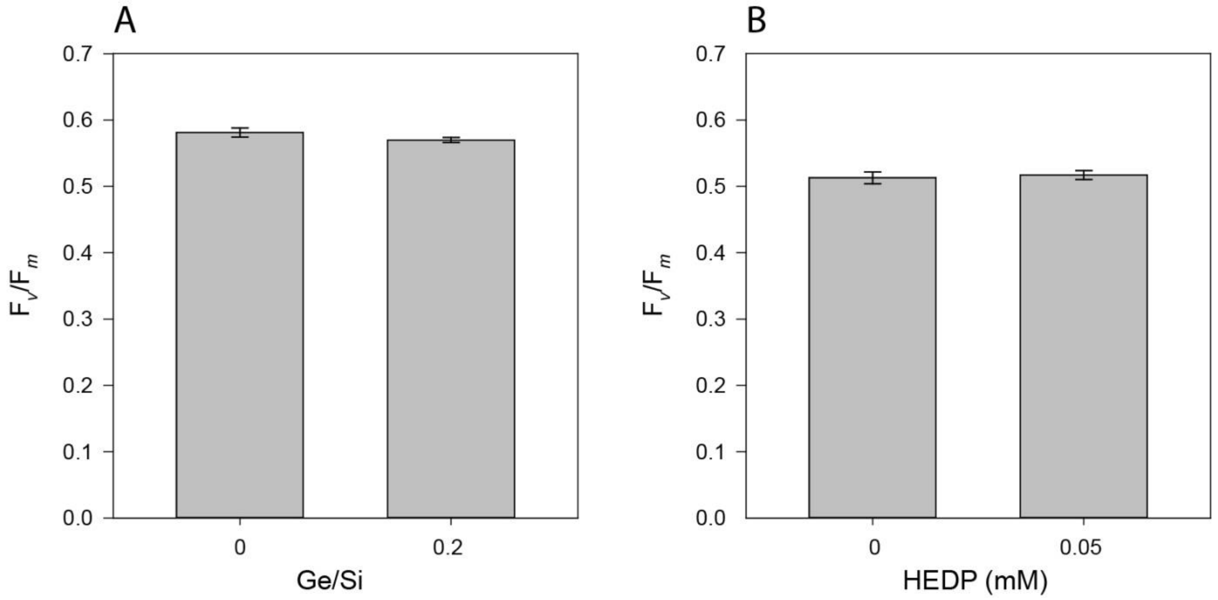

Fig. S3: Photosynthetic efficiency following disruption of calcification

A) The photosynthetic efficiency of photosystem II (quantum yield, F_v/F_m) following treatment of *C. braarudii* cells with 20 µM Ge (0.2 Ge/Si) B) Measurement of F_v/F_m for *C. braarudii* at 0.05 mM HEDP for 72 h. No significant difference was observed in either treatment (p=>0.05, n=3, two-tailed *t*-test). Error bars denote standard error.

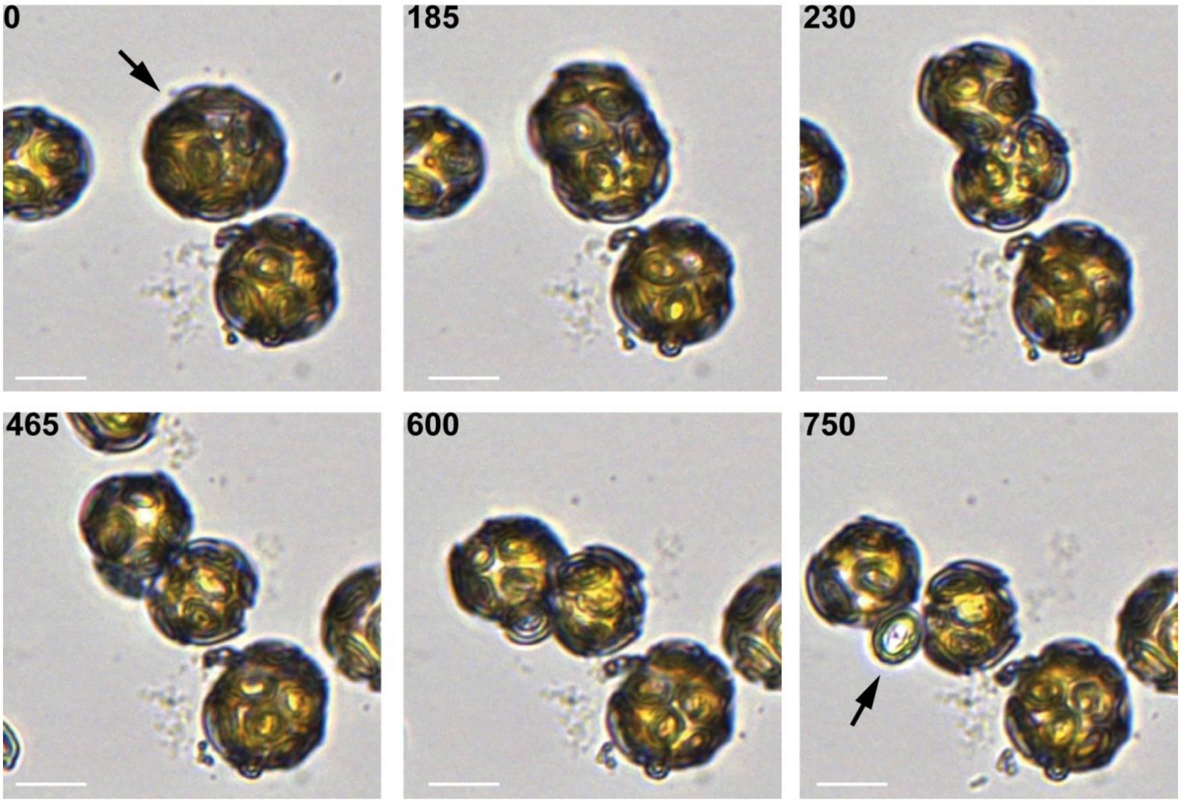

Fig. S4: Time-lapse microscopy of cell division in *C. braarudii*

Time-lapse imaging of *C. braarudii* undergoing cell division recorded over 16 h in the dark (cells were illuminated for 100 ms every 5 minutes in order to record an image). At the onset of cell division, the cell begins to elongate (185 min) and the coccoliths move flexibly on the cell surface to maintain a complete coccosphere. As the cell divides, the coccosphere rearranges to ensure both daughter cells are fully covered following division (230-465 min). The cells separate following rearrangement of the coccospheres (600-750 min) and a complete coccolith is dislodged prior to separation of the two daughter cells (arrowed). Frame labels denote minutes passed and scale bars denote 15 μm.

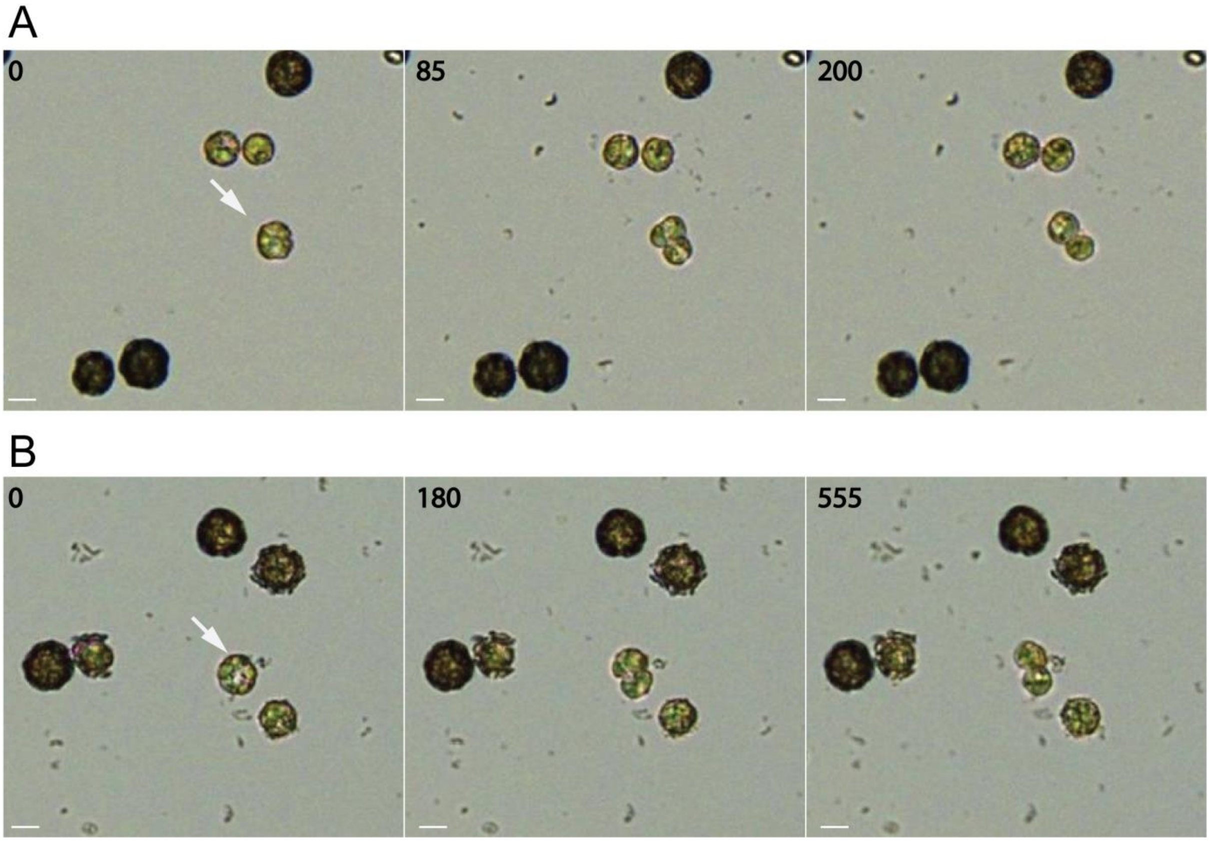

Fig. S5: Cell division can occur in the absence of a coccosphere

C. braarudii cells were decalcified using ASW minus Ca^{2+} pH 7.0 for 1 h. This timescale results in significant numbers of fully decalcified cells. Time-lapse images were recorded for 16 h in the dark (17°C) to observe cell division of the decalcified cells. A, B) examples of fully decalcified cells undergoing cell division (arrows). The cells are able to divide when fully decalcified but remain in pairs after cytokinesis takes place. Frame labels denote minutes passed and scale bars denote 15 µm.

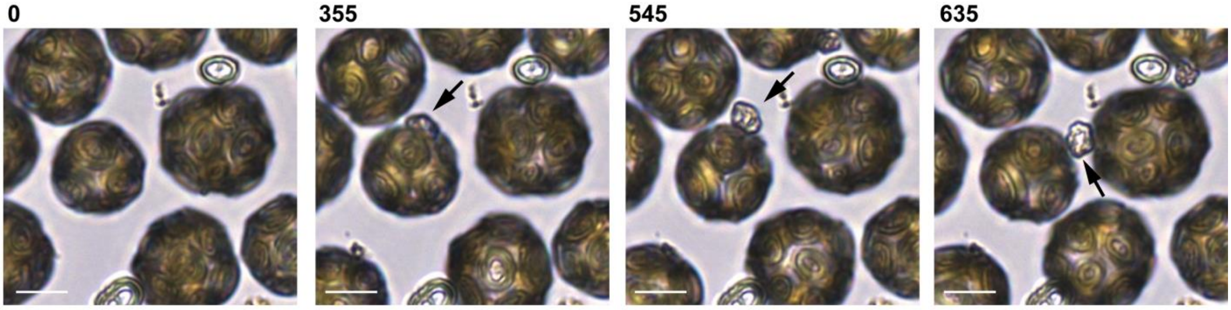

Fig. S6: Malformed coccolith production in Ge-treated cells

C. braarudii cells were incubated in Ge/Si 0.2 (10 µM Si). Time-lapse images were recorded over 16 h in the light (17°C) to observe the effects of Ge. Cells treated with Ge are initially fully calcified. In the example shown, a cell produces a highly malformed coccolith 6 h after addition of Ge (arrow). The malformed coccolith is unable to integrate into the coccosphere and is discarded into the surrounding media. Frame labels denote minutes passed and scale bars denote 10 µm.

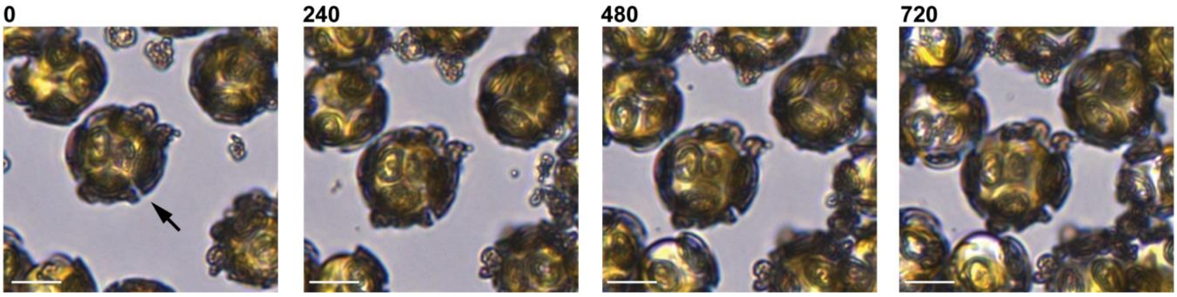

Fig. S7: Ge-treated cells exhibit a progressive disruption of the coccosphere as cell volume increases

Time-lapse LM footage was recorded over 12 h in the light (17°C) to observe the effects of Ge on coccolith production. *C. braarudii* cells were grown in Ge/Si 0.2 (10 µM Si) for 4 d. In control cells, coccoliths are continuously produced and incorporated into the coccosphere as volume of the growing cell increases, ensuring the cell remains covered by a single layer of coccoliths. Ge-treated cells also continue to calcify and exhibit an increase in cell volume during the 12 h time-lapse period. However, as the malformed coccoliths are not integrated into the coccosphere, the coccosphere covers proportionately less of the cell body as the cell expands. As a result, the disruption to the coccosphere is visibly greater at the end of time lapse (720 min) the compared to the start (T 0) (see arrowed cell). Frame labels denote minutes passed and scale bars denote 10 µm.

Inhibitor		24 h		48 h		72 h	
		Control	Inhibitor	Control	Inhibitor	Control	Inhibitor
**Low Ca^{2+}**	Incomplete (%)	9.42*	8.49	12.53	20.69	6.56	29.75
	Total Coccoliths Scored	456	412	431	435	457	437
**Ge**	Malformed (%)	0.00	4.03	0.76	13.74	0.38	11.08**
	Total Coccoliths Scored	552	521	526	502	526	469
**HEDP**	Malformed (%)	0.41	3.24	0.21	15.40	0.00	25.00
	Total Coccoliths Scored	487	432	475	435	471	471

Incomplete: where calcification of the coccolith has begun but stopped before completion
Malformed: coccoliths exhibiting gross defects in morphology, e.g. irregular shaping of the calcite crystals

* *C. braarudii* cells grown in ASW typically exhibit an elevated level of incomplete coccoliths relative to cells grown in natural seawater.

** Coccoliths generated during Ge treatment are highly malformed and often fail to integrate into the coccosphere.

Table S1: Disruption of calcification in *C. braarudii* by low Ca^{2+}, HEDP or Ge, determined as the percentage of incomplete or malformed coccoliths in the coccosphere.

Family	Species	Isolates often become partially- or non-calcified in laboratory culture?*	Examples of non-calcified diploid strains in culture collections	Reports of non-calcified strains in literature (see supplementary references)
Calcidiscaceae	*Calcidiscus leptoporus*	NO	None	
	Calcidiscus quadriperforatus	NO	None	
	Oolithotus fragilis	NO	None	
	Umbilicosphaera sibogae	NO	None	
	Umbilicosphaera foliosa	NO	None	
	Umbilicosphaera hulburtiana	NO**	None	
Coccolithaceae	*Coccolithus pelagicus*	NO	None	
	Coccolithus braarudii	NO	None	
Hymenomonadaceae	*Ochrosphaera neapolitana*	YES	CCAP932/1; RCC1358; RCC1365	
	Hymenomonas coronata	YES	RCC1337	
Pleurochrysidaceae	*Chrysotila carterae*	YES	CCMP645; RCC1402	1,2
	Chrysotila dentata	YES	CCAP904/1; RCC1394	
	Chrysotila pseudoroscoffensis	YES	CCAP912/1; CCAP913/2; CCAP913/3; CCAP961/3	
Helicosphaeraceae	*Syracosphaera pulchra*	NO	None	
	Helicosphaera carteri	NO	None	
Pontosphaeraceae	*Scyphosphaera apsteinii*	NO	None	
Noelaerhabdaceae	*Gephyrocapsa oceanica*	NO	None	
	Emiliania huxleyi	YES	CCMP370; CCMP373; CCMP374; CCMP379; CCMP1280; CCMP1516; CCMP2090	3,4

*Non-calcified strains are defined as diploid strains which can persist in a non-calcified state without any adverse effects on cell fitness; calcified strains are those in which healthy diploid cultures do not exhibit non-calcified cells.

**A single isolate (strain RCC1474) was observed to grow in a non-calcified state for a prolonged period, although it has subsequently regained a calcified state (I. Probert - personal communication).

Table S2: The calcification status of diploid coccolithophore strains in algal culture collections

Supplementary References

1 Marsh, M. E. & Dickinson, D. P. Polyanion-mediated mineralization — mineralization in coccolithophore (*Pleurochrysis carterae*) variants which do not express PS2, the most abundant and acidic mineral-associated polyanion in wild-type cells. *Protoplasma* **199**, 9-17, doi:10.1007/bf02539801 (1997).

2 Marsh, M. E. Biomineralization in Coccolithophores. *Biomineralization: Progress in Biology, Molecular Biology and Application* (2006).

3 Klaveness, D. *Coccolithus Huxleyi (Lohmann) Kamptner: Morphological Investigations on the Vegetative Cell and the Process of Coccolith Formation*. (1972).

4 Paasche, E. A review of the coccolithophorid *Emiliania huxleyi* (Prymnesiophyceae), with particular reference to growth, coccolith formation, and calcification-photosynthesis interactions. *Phycologia* **40**, 503-529, doi:10.2216/i0031-8884-40-6-503.1 (2001).

Video S1: Cell division in *C. braarudii*

Time-lapse light microscopy imaging of *C. braarudii* undergoing cell division recorded over 7.5 h in the dark (cells were illuminated for 100 ms every 5 minutes in order to record an image). Frame labels denote minutes passed.
